# Correlation of silent brain infarcts and leukoaraiosis in middle-aged ischemic stroke patients: a retrospective study

**DOI:** 10.3389/fneur.2024.1430231

**Published:** 2024-08-21

**Authors:** Mohammad Fathi Abdulsalam, Nour Shaheen, Ahmed Shaheen, Yasmeen Jamal Alabdallat, Abdelraouf Ramadan, Mostafa Meshref, Fathy Mahmoud Mansour, Elsayed Abed, Abdel-Ghaffar I. Fayed, Mohamed Ahmed Zaki, Ahmad F. El-Adawy, Oliver Flouty, Mohamed Hamed

**Affiliations:** ^1^Department of Neurology, Faculty of Medicine, Al-Azhar University, Cairo, Egypt; ^2^Alexandria Faculty of Medicine, Alexandria University, Alexandria, Egypt; ^3^Faculty of Medicine, Hashemite University, Zarqa, Jordan; ^4^Kasr Alainy Faculty of Medicine, Cairo University, Cairo, Egypt; ^5^Department of Neurosurgery and Brain Repair, University of South Florida, Tampa, FL, United States

**Keywords:** cerebrovascular disease, ischemic stroke, leukoaraiosis, silent brain infarcts, silent lacunar infarcts, metabolic syndrome

## Abstract

**Background:**

Cerebrovascular diseases of the brain are usually defined by transient ischemic attacks and strokes. However, they can also cause brain injuries without neurological events. Silent brain infarcts (SBI) and leukoaraiosis are symptoms of both vascular and neurological abnormalities. This study aims to investigate the association between SBI, leukoaraiosis, and middle-aged patients with ischemic stroke.

**Methods:**

A single-center retrospective study of 50 middle-aged, ischemic stroke patients were studied from November 2022 and May 2023. The patients were divided into two groups based on the presence or absence of leukoaraiosis. History taking, physical examination, brain CT scan, and MRI were all part of the diagnostic process. Metabolic syndrome (MetS) was also assessed through various factors. The statistical analysis included descriptive statistics, logistic regression analysis, and chi-square test.

**Results:**

Out of the cohort comprising 50 patients, characterized by a mean age of 52.26 years (SD 5.29), 32 were male, constituting 64% of the sample. Among these patients, 26 individuals exhibited leukoaraiosis, with 17 of them (65.4%) also presenting with SBI. Moreover, within this cohort, 22 patients were diagnosed with MetS, representing 84.6% of those affected. The Multivariate logistic regression analysis showed a strong and independent association between leukoaraiosis and SBI. Individuals with leukoaraiosis were nearly five times more likely to have SBI compared to those without leukoaraiosis.

**Conclusion:**

The study highlights leukoaraiosis as a significant risk factor for SBI, alongside MetS. Advanced imaging techniques have facilitated their detection, revealing a higher prevalence among stroke patients, particularly associated with age and hypertension. Further research is needed to fully understand their complex relationship and develop better management strategies for cerebrovascular diseases, ultimately improving patient outcomes.

## Introduction

Historically, cerebrovascular disease of the brain has been defined by the symptoms and signs of transient ischemic attack or stroke. However, neuropathological studies in highly selected populations have revealed that vascular disease can cause brain injury in the absence of these acute neurological events. The advent of advanced brain-imaging techniques, such as computerized tomography (CT) and Magnetic resonance imaging (MRI), has allowed similar observations to be made in patient groups and healthy individuals, necessitating a reconsideration of the definition of cerebrovascular disease (1, 2). Signs of cerebral small vessel disease on conventional MRI include leukoaraiosis, recent subcortical lacunar infarcts (clinically symptomatic), lacunes (clinically silent), cerebral microbleeds, prominent perivascular spaces, and cerebral atrophy (3). These brain infarcts, while often asymptomatic, demand increased attention to mitigate the deleterious effects of vascular disease in the brain. Silent brain infarctions (SBIs) comprise two subtypes: lacunar and non-lacunar, resulting from small perforating artery occlusion and embolism or athero-sclerotic stenosis, respectively. The advancement of MRI technology enables the distinction between these subtypes (4–6). Therefore, exploring the distinct risk factors between the two subtypes, especially in the case of SBI, could lead to the development of specific prevention strategies, particularly for middle-aged individuals. Hypertension (HTN), apart from age, is the most widely accepted risk factor associated with SBI. Furthermore, the consistent correlation between hypertension and these infarcts suggests a critical role for hypertensive small-vessel disease in their pathogenesis (7). However, further research is necessary to better define the association between hypertension and brain infarcts, particularly in terms of preventing SBI through effective hypertension control. SBI and leukoaraiosis (LA) are intricate cerebral manifestations that have garnered considerable attention due to their association with diverse vascular and metabolic abnormalities. Hence, comprehending the intricate relationship between these cerebral alterations and MetS is of paramount importance for elucidating their underlying mechanisms and devising effective prevention and management strategies. Leukoaraiosis was observed through MRI and manifests as increased signal intensity in the white matter, often attributed to small vessel disease and pathological processes such as demyelination, gliosis, and vessel lipo hyalinosis (8). Conversely, SBIs denote brain tissue damage resulting from inadequate blood supply without acute neurological symptoms. Though often asymptomatic, SBIs pose a substantial risk for future stroke and cognitive decline (9). MetS plays a pivotal role in the development of LA and SBIs, operating through mechanisms such as vascular dysfunction, inflammation, insulin resistance, and dyslipidemia (10). The diagnosis of LA and SBIs primarily relies on MRI techniques, with fluid-attenuated inversion recovery (FLAIR) imaging sequences commonly employed to detect and assess the extent of white matter changes (11). Epidemiological data indicate a higher prevalence of LA and SBIs with advancing age, affecting a significant proportion of individuals over 65 years. This research aims to investigate the association between LA, SBIs, and middle-aged patients with ischemic stroke. The study focuses on middle-aged stroke patients to address the critical period in stroke epidemiology, capture a substantial portion of stroke cases in a relatively younger age group, identify early risk factors and pathophysiological mechanisms, and provide clinically relevant insights for healthcare providers in terms of risk stratification, diagnostics, and treatment strategies.

## Methodology

### Study design and setting

We conducted a retrospective single-center cohort study involving middle-aged ischemic stroke patients admitted to our university hospitals or followed up in our outpatient clinic between November 2022 and May 2023.

### Participants selection

The study included middle-aged (35–64 years) (12) patients with ischemic stroke, who were divided into two groups. Group 1 consisted of patients with ischemic stroke associated with leukoaraiosis, while Group 2 consisted of patients with ischemic stroke not associated with leukoaraiosis.

The patients were diagnosed with ischemic stroke through a comprehensive process involving history taking, physical examination (including general and neurological examination), and a radiologic study using a brain CT scan.

To further evaluate the patients, the brain’s magnetic resonance imaging (MRI) was performed to diagnose and grade leukoaraiosis. The MRI examinations were carried out using a 1.5 Tesla superconducting magnet system. The imaging protocol included T2-weighted, T1-weighted, and fluid-attenuated inversion recovery (FLAIR) images. Leukoaraiosis was defined as a white matter lesion showing hyperintensity on T2-weighted and FLAIR images without prominent hypointensity on TI-weighted images. The grading of leukoaraiosis was done according to the Atherosclerosis Risk in Communities Study (ARIC) criteria (13–15).

MetS assessment involves the evaluation of various factors, including impaired fasting glucose (IFG), elevated blood pressure (BP), hypertriglyceridemia (hyper-TG), low high-density lipoprotein cholesterol (HDL-C), and waist circumference. IFG was defined as a fasting glucose level of 110 mg/dL or higher, and elevated BP was determined by systolic BP of 130 mm Hg or higher and diastolic BP of 85 mm Hg or higher. Hyper-TG was identified based on serum triglyceride levels of 150 mg/dL or higher, while low HDL-C was defined as serum HDL-C levels below 40 mg/dL for men and below 50 mg/dL for women. Waist circumference was measured at a specific anatomical point (16).

### Data collection

The data collected for this study included: (1) Demographics: Age and gender of the participants. (2) Medical Conditions: Presence of SBI and leukoaraiosis (3) Metabolic Syndrome (MetS): Presence or absence of MetS, as well as its individual components including Elevated blood pressure (BP), Impaired fasting glucose (IFG), Low high-density lipoprotein cholesterol (HDL-C), Hypertriglyceridemia (Hyper-TG), Large waist circumference (WC) 4. Atherosclerosis Risk in Communities (ARIC) Grades: The severity of atherosclerosis was graded as none, minimal, mild, moderate, or severe.

### Statistical analysis

The statistical analysis of the study involved the use of descriptive statistics, such as mean, standard deviation (SD), frequencies (N), and percentages (%), to summarize the data. MetS component conditions were treated as dichotomous variables based on NCEP/ATP III-defined cut points. The three grades of leukoaraiosis (severe, moderate, and mild) were combined due to a small number of subjects with those specific grades. The chi-square test examined statistically significant relationships between different qualitative data. Logistic regression analysis was performed to estimate the association between each variable and leukoaraiosis while controlling for other variables; it was expressed using odds ratio (OR) and 95% CI. A *p*-value of less than 0.05 was considered significant, while a p-value of less than 0.01 was considered highly significant. R (version 4.3.1)was used for all analyses.

### Sample size

The study included 50 middle-aged ischemic stroke patients. This sample size was determined based on the availability of data from our hospital records during the study period. A post-hoc power analysis was performed to assess the study’s power to detect a statistically significant association between leukoaraiosis and silent brain infarcts (SBI) given the observed effect size.

## Results

### Participant characteristics

The study included 50 ischemic stroke patients with a mean age of 52.26 (± 5.29) years with 32 (64%) male patients. The participants were divided into two groups: a control group (26 participants) without leukoaraiosis and an experimental group (24 participants) with leukoaraiosis. The median age was higher in patients with SBI compared to the SBI-negative group (*p* = 0.149), with males’ predominance in the SBI group (75%, *p* = 0.207).

### Association of silent brain infarcts with leukoaraiosis and other risk factors

MetS was significantly more common in the SBI-positive group (87.5%) compared to the negative group (38.5%, *p* = 0.001). HTN and impaired fasting glucose (IFG) were also significantly more common in the SBI-positive group (*p* = 0.001 and *p* < 0.001, respectively). The population shows that 65.4% of those without SBI had no atherosclerosis (grade None) compared to only 29.2% of those with SBI. The presence of elevated blood pressure (BP), IFG, hypertriglyceridemia (hyper-TG), large waist circumference (WC), leukoaraiosis, and SBI were all significantly higher in individuals with MetS than those without MetS. Regarding the ARIC grade, individuals with MetS had a higher prevalence of leukoaraiosis than those without MetS (*p* = 0.002). The median age of those with leukoaraiosis (55 years) was significantly higher than those without (50 years); (*p* = 0.001). In addition, the percentage of individuals with MetS, HTN, IFG, and SBI was significantly higher in the group with leukoaraiosis compared to the group without leukoaraiosis. The demographics of the included participants are shown in Table 1.

**Table 1:** Demographics of the included participants.

		Frequency (*n*^a^)	Percentage (%^b^)
**Characteristics (*n* = 50)**			
Age, Mean (SD)		52.26 (5.29)	
Gender	Male	32	64
	Female	18	36
SBI		24	48
Leukoaraiosis		26	52
MetS		31	62
MetS Components	Elevated BP^c^	31	62
	IFG^d^	28	56
	Low HDL-C^e^	25	50
	Hyper-TG^f^	17	34
	Large WC^g^	24	48
**The ARIC grades** ^**h**^			
None		24	48
Minimal		10	20
Mild		6	12
Moderate		6	12
Severe		4	8
**SBI**			
	**Negative**	**Positive**	***P*-value**
*N*	26	24	
Age, Median [IQR]	51.50 [49.25, 54.75]	55.00 [48.75, 58.25]	0.149
Gender (M), *N* (%)	14 (53.8)	18 (75.0)	0.207
MetS, *N* (%)	10 (38.5)	21 (87.5)	0.001^*^
Elevated BP^c^	8 (30.8)	20 (83.3)	0.001^*^
IFG^d^	6 (23.1)	19 (79.2)	<0.001^*^
Low HDL-C^e^	9 (34.6)	8 (33.3)	1
Hyper-TG^f^	11 (42.3)	13 (54.2)	0.579
Large WC^g^	16 (61.5)	19 (79.2)	0.294
The ARIC grades^h^, *N* (%)			0.019^*^
None	17 (65.4)	7 (29.2)	-
Minimal	6 (23.1)	4 (16.7)	-
Mild	2 (7.7)	4 (16.7)	-
Moderate	1 (3.8)	5 (20.8)	-
Severe	0 (0.0)	4 (16.7)	-
Leukoaraiosis, *N* (%)	9 (34.6)	17 (70.8)	0.023^*^
**MetS**			
	**Negative**	**Positive**	***P*-value**
*N*	19	31	
Age, Median [IQR]	51.00 [49.00, 54.50]	54.00 [49.50, 58.00]	0.182
Gender (M), *N* (%)	10 (52.6)	22 (71.0)	0.314
Elevated BP^c^	4 (21.1)	24 (77.4)	<0.001^*^
IFG^d^	4 (21.1)	21 (67.7)	0.004^*^
Low HDL-C^e^	3 (15.8)	14 (45.2)	0.069
Hyper-TG^f^	3 (15.8)	21 (67.7)	0.001^*^
Large WC^g^	10 (52.6)	25 (80.6)	0.075
The ARIC grades^h^, *N* (%)			0.013^*^
None	15 (78.9)	9 (29.0)	-
Minimal	2 (10.5)	8 (25.8)	-
Mild	1 (5.3)	5 (16.1)	-
Moderate	0 (0.0)	6 (19.4)	-
Severe	1 (5.3)	3 (9.7)	-
Leukoaraiosis, *N* (%)	4 (21.1)	22 (71.0)	0.002^*^
SBI	3 (15.8)	21 (67.7)	0.001^*^
**Leukoaraiosis**			
	**Negative**	**Positive**	***P*-value**
*N*	24	26	
Age, Median [IQR]	50.00 [47.00, 53.00]	55.00 [51.50, 58.75]	0.001^*^
Gender (M), *N* (%)	14 (58.3)	18 (69.2)	0.612
MetS, *N* (%)	9 (37.5)	22 (84.6)	0.002^*^
Elevated BP^c^	8 (33.3)	20 (76.9)	0.005^*^
IFG^d^	7 (29.2)	18 (69.2)	0.011^*^
Low HDL-C^e^	7 (29.2)	10 (38.5)	0.693
Hyper-TG^f^	9 (37.5)	15 (57.7)	0.252
Large WC^g^	16 (66.7)	19 (73.1)	0.853
SBI	7 (29.2)	17 (65.4)	0.023^*^

^a^Frequency, ^b^Percentage, ^c^Blood Pressure, ^d^Impaired Fasting Glucose, ^e^High-density lipoprotein-cholesterol, ^f^Triglyceride, ^g^Waist Circumflex, ^h^Atherosclerosis Risk in Communities grades, SBI, Silent brain infarct; MetS, Metabolic syndrome.

The study found that 48% of patients had SBI, and 52% had leukoaraiosis. The chi-square test revealed that the association between leukoaraiosis and SBI was not statistically significant (*p* = 0.13). In terms of sex, 36% of patients were female, and 64% were male. The chi-square test revealed a significant association between sex and SBI (*p* = 0.013), with a higher percentage of male patients having silent brain infarction than female patients (Table 2).

**Table 2:** Summary of the association between leukoaraiosis and silent brain infarction with respect to demographic and metabolic variables, metabolic syndrome components, and ARIC grades.

**Leukoaraiosis**		**SBI**					
		**Negative**		**Positive**		**Total**	
		***N*** ^**a**^	**%** ^**b**^	***N*** ^**a**^	**%** ^**b**^	***N*** ^**a**^	**%** ^**b**^
Negative		17	34	7	14	24	48
Positive		9	18	17	34	26	52
Total		26	52	24	48	50	100
Chi-square	X^2^	2.424					
	*P*-value	0.149					
**Sex**		**SBI**					
		**Negative**		**Positive**		**Total**	
		***N*** ^**a**^	**%** ^**b**^	***N*** ^**a**^	**%** ^**b**^	***N*** ^**a**^	**%** ^**b**^
Female		12	24	6	12	18	36
Male		14	28	18	36	32	64
Total		26	52	24	48	50	100
Chi-square	X^2^	6.559					
	*P*-value	0.013*					
**MetS**		**SBI**					
		**Negative**		**Positive**		**Total**	
		***N*** ^**a**^	**%** ^**b**^	***N*** ^**a**^	**%** ^**b**^	***N*** ^**a**^	**%** ^**b**^
Negative		16	32	3	6	9	38
Positive		10	20	21	42	31	62
Total		26	52	24	48	50	100
Chi-square	X^2^	12.738					
	*P*-value	0.000*					
**MetS Components**		**SBI**					
**Elevated BP** ^**c**^		**Negative**		**Positive**		**Total**	
		***N*** ^**a**^	**%** ^**b**^	***N*** ^**a**^	**%** ^**b**^	***N*** ^**a**^	**%** ^**b**^
Negative		18	36	4	8	22	44
Positive		8	16	20	40	28	56
Total		26	52	24	48	50	100
Chi-square	X^2^	13.994					
	*P*-value	0.000*					
**IFG** ^**d**^		**Negative**		**Positive**		**Total**	
		***N*** ^**a**^	**%** ^**b**^	***N*** ^**a**^	**%** ^**b**^	***N*** ^**a**^	**%** ^**b**^
Negative		20	40	5	10	25	50
Positive		6	12	19	38	25	50
Total		26	52	24	48	50	100
Chi-square	X^2^	15.705					
	*P*-value	0.000*					
**Low HDL-C** ^**e**^		**Negative**		**Positive**		**Total**	
		***N*** ^**a**^	**%** ^**b**^	***N*** ^**a**^	**%** ^**b**^	***N*** ^**a**^	**%** ^**b**^
Negative		17	34	16	32	33	66
Positive		9	18	8	16	17	34
Total		26	52	24	48	50	100
Chi-square	X^2^	0.009					
	*P*-value	1.000					
**Hyper-TG** ^**f**^		**Negative**		**Positive**		**Total**	
		***N*** ^**a**^	**%** ^**b**^	***N*** ^**a**^	**%** ^**b**^	***N*** ^**a**^	**%** ^**b**^
Negative		15	30	11	22	26	52
Positive		11	22	13	26	24	48
Total		26	52	24	48	50	100
Chi-square	X^2^	0.703					
	*P*-value	0.572					
**Large WC** ^**g**^		**Negative**		**Positive**		**Total**	
		***N*** ^**a**^	**%** ^**b**^	***N*** ^**a**^	**%** ^**b**^	***N*** ^**a**^	**%** ^**b**^
Negative		10	20	5	10	15	30
Positive		16	32	19	38	35	70
Total		26	52	24	48	50	100
Chi-square	X^2^	1.847					
	*P*-value	9.224					
**The ARIC**^**h**^ **grades**		**SBI**					
		**Negative**		**Positive**		**Total**	
		***N*** ^**a**^	**%** ^**b**^	***N*** ^**a**^	**%** ^**b**^	***N*** ^**a**^	**%** ^**b**^
None		17	34	7	14	24	48
Minimal		6	12	4	8	10	20
Mild		2	4	4	8	6	12
Moderate		1	2	5	10	6	12
Severe		0	0	4	8	4	8
Total		26	52	24	48	50	100
Chi-square	X^2^	11.839					
	*P*-value	0.019*					
**SBI**		**Leukoaraiosis**					
		**Negative**		**Positive**		**Total**	
		***N*** ^**a**^	**%** ^**b**^	***N*** ^**a**^	**%** ^**b**^	***N*** ^**a**^	**%** ^**b**^
Negative		17	34	9	18	26	52
Positive		70	14	17	34	24	48
Total		24	48	26	52	50	100
Chi-square	X^2^	6.559					
	*P*-value	0.13					
**Sex**		**Leukoaraiosis**					
		**Negative**		**Positive**		**Total**	
		***N*** ^**a**^	**%** ^**b**^	***N*** ^**a**^	**%** ^**b**^	***N*** ^**a**^	**%** ^**b**^
Female		10	20	8	16	18	36
Male		14	28	18	36	32	64
Total		24	48	26	52	50	100
Chi-square	X^2^	0.643					
	*P*-value	0.557					
**MetS**		**Leukoaraiosis**					
		**Negative**		**Positive**		**Total**	
		***N*** ^**a**^	**%** ^**b**^	***N*** ^**a**^	**%** ^**b**^	***N*** ^**a**^	**%** ^**b**^
Negative		15	30	4	8	19	38
Positive		9	18	22	44	31	62
Total		24	48	26	52	50	100
Chi-square	X^2^	11.759					
	*P*-value	0.001*					
**MetS Components**		**Leukoaraiosis**					
**Elevated BP** ^**c**^		**Negative**		**Positive**		**Total**	
		***N*** ^**a**^	**%** ^**b**^	***N*** ^**a**^	**%** ^**b**^	***N*** ^**a**^	**%** ^**b**^
Negative		16	32	6	12	22	44
Positive		8	16	20	40	28	56
Total		24	48	26	52	50	100
Chi-square	X^2^	9.624					
	*P*-value	0.004*					
**IFG** ^**d**^		**Negative**		**Positive**		**Total**	
		***N*** ^**a**^	**%** ^**b**^	***N*** ^**a**^	**%** ^**b**^	***N*** ^**a**^	**%** ^**b**^
Negative		17	34	8	16	25	50
Positive		7	14	18	36	25	50
Total		24	48	26	52	50	100
Chi-square	X^2^	8.013					
	*P*-value	0.010*					
**Low HDL-C** ^**e**^		**Negative**		**Positive**		**Total**	
		***N*** ^**a**^	**%** ^**b**^	***N*** ^**a**^	**%** ^**b**^	***N*** ^**a**^	**%** ^**b**^
Negative		17	34	16	32	33	66
Positive		7	14	10	20	17	34
Total		24	48	26	52	50	100
Chi-square	X^2^	0.480					
	*P*-value	0.559					
**Hyper-TG** ^**f**^		**Negative**		**Positive**		**Total**	
		***N*** ^**a**^	**%** ^**b**^	***N*** ^**a**^	**%** ^**b**^	***N*** ^**a**^	**%** ^**b**^
Negative		15	30	11	22	26	52
Positive		9	18	15	30	24	48
Total		24	48	26	52	50	100
Chi-square	X^2^	2.039					
	*P*-value	0.171					
**Large WC** ^**g**^		**Negative**		**Positive**		**Total**	
		***N*** ^**a**^	**%** ^**b**^	***N*** ^**a**^	**%** ^**b**^	***N*** ^**a**^	**%** ^**b**^
Negative		8	16	7	14	15	30
Positive		16	32	19	38	35	70
Total		24	48	26	52	50	100
Chi-square	X^2^	0.244					
	*P*-value	0.760					
**The ARIC**^**h**^ **grades**		**Leukoaraiosis**					
		**Negative**		**Positive**		**Total**	
		***N*** ^**a**^	**%** ^**b**^	***N*** ^**a**^	**%** ^**b**^	***N*** ^**a**^	**%** ^**b**^
None		24	48	0	0	24	48
Minimal		0	0	10	20	10	20
Mild		0	0	6	12	6	12
Moderate		0	0	6	12	6	12
Severe		0	0	4	8	4	8
Total		24	48	26	52	50	100
Chi-square	X^2^	50.000					
	*P*-value	0.000*					

^*^p < 0.05. ^a^Frequency, ^b^Percentage, ^c^Blood Pressure, ^d^Impaired Fasting Glucose, ^e^High-density lipoprotein-cholesterol, ^f^Triglyceride, ^g^Waist Circumflex, ^h^ Atherosclerosis Risk in Communities grades. SBI, Silent brain infarct; MetS, Metabolic syndrome.

### Association of leukoaraiosis with MetS and its components

In terms of MetS, 62% of patients had MetS, and the chi-square test revealed a significant association between MetS and SBI (*p* = 0.000). Patients with MetS had a higher percentage of SBI than patients without MetS. Similarly, MetS components, including elevated blood pressure, impaired fasting glucose, and large waist circumference, were significantly associated with silent brain infarction. The ARIC grades also showed a significant association with SBI (*p* = 0.019), with patients in higher ARIC grades having a higher percentage of silent brain infarction. Overall, the study found that SBI was significantly associated with sex, MetS, MetS components, and the ARIC grades. However, there was no significant association between leukoaraiosis and SBI.

The results indicate that there was a significant association between MetS and leukoaraiosis (*p* = 0.001), as well as between some of its components (elevated BP and IFG) and leukoaraiosis (*p* = 0.004 and *p* = 0.010, respectively). However, there was no significant association between sex or large WC and leukoaraiosis (Table 2).

### Severity of leukoaraiosis and MetS

The severity of leukoaraiosis was significantly associated with the presence of MetS, as well as its components, with a higher proportion of participants with more severe leukoaraiosis having MetS or elevated BP or IFG (Table 2).

### Multivariable analysis of SBI and leukoaraiosis

The results of the analysis showed that leukoaraiosis is significantly associated with SBI with an unadjusted odds ratio (OR) of 4.587 (*p* = 0.012). The adjusted OR for the other predictors does not change the significance of the association between leukoaraiosis and SBI, indicating that this association is independent of the other predictors in the model. Therefore, the results emphasize the importance of considering SBI as a risk factor for leukoaraiosis. The results suggest that MetS, elevated BP, IFG, and leukoaraiosis are significantly associated with an increased risk of SBI (OR = 4.587 [0.985–8.190]; *p* = 0.012). Therefore, these findings suggest that individuals with leukoaraiosis may be at an increased risk for SBI and may benefit from closer monitoring and potential interventions to reduce their risk. The OR and 95% confidence intervals for the association between various risk factors of SBI and leukoaraiosis are shown in Table 3.

**Table 3:** Multivariate logistic regression analysis showing predictors of **(A)** Silent Brain Infarcts, **(B)** MetS, and **(C)** Leukoaraiosis.

Dependent variable	Independent variable	Unadjusted OR^a^	95% confidence interval (CI)	*p*-value
**A**				
SBI	Age	1.081	(−1.003, 3.165)	0.171
	Gender	2.571	(−0.064, 5.206)	0.124
	MetS^b^	11.200	(7.108, 15.292)	0.001^*^
	Elevated BP^c^	11.250	(7.336, 15.164)	0.000^*^
	IFG^d^	12.667	(8.761, 16.573)	0.000^*^
	Low HDL-C^e^	0.944	(−2.624, 4.512)	0.924
	Hyper-TG^f^	1.612	(−1.861, 5.084)	0.403
	Large WC^g^	2.375	(−0.389, 5.139)	0.179
	Leukoaraiosis	4.587	(0.985, 8.190)	0.012^*^
**B**				
MetS^b^	Age	1.25944	(0.207227, 7.654423)	0.002^*^
	Gender	1.607143	(0.208937, 12.358964)	0.42
	MetS^b^	9.166667	(2.297217, 36.614768)	0.001^*^
	Elevated BP^c^	6.666667	(1.930124, 22.978844)	0.002^*^
	IFG^d^	5.464286	(1.465766, 20.441014)	0.006^*^
	Low HDL-C^e^	1.517857	(0.312961, 7.364701)	0.489
	Hyper-TG^f^	2.272727	(0.506725, 10.178785)	0.156
	Large WC^g^	1.357143	(0.324471, 5.679855)	0.621
	Silent Lacunar Infarcts	4.587302	(1.329704, 15.810814)	0.01^*^
**C**				
Leukoaraiosis	Age	1.25944	(0.181007, 2.337873)	0.002^*^
	Gender	1.607143	(−2.862666, 6.076952)	0.423
	MetS^b^	9.166667	(5.281997, 13.05134)	0.001^*^
	Elevated BP^c^	6.666667	(2.962831, 10.3705)	0.002^*^
	IFG^d^	5.464286	(1.853614, 9.074957)	0.006^*^
	Low HDL-C^e^	1.517857	(−2.125023, 5.160737)	0.489
	Hyper-TG^f^	2.272727	(−0.774381, 5.319834)	0.156
	Large WC^g^	1.357143	(−2.428864, 5.14315)	0.621
	Silent Lacunar Infarcts	4.587302	(0.812214, 8.36239)	0.01^*^

^*^p < 0.05. ^a^Odds Ratio, ^b^Metabolic Syndrome, ^c^Blood Pressure, ^d^Impaired Fasting Glucose, ^e^High-density lipoprotein-cholesterol, ^f^Triglyceride, ^g^Waist Circumflex, ^h^Atherosclerosis Risk in Communities grades. SBI, Silent brain infarct; MetS, Metabolic syndrome.

## Discussion

The principal finding of the study is the significant association between leukoaraiosis and SBI, indicating that leukoaraiosis should be considered a significant risk factor for SBI. Additionally, MetS, particularly its components such as elevated blood pressure and impaired fasting glucose, showed a strong association with both leukoaraiosis and SBI. These findings underscore the importance of recognizing and managing these risk factors to potentially reduce the incidence of SBI and their associated complications.

SBIs and leukoaraiosis are common findings in patients with stroke. Several studies aimed at estimating the percentage of patients with SBIs or leukoaraiosis that presented with stroke. Putaala et al. found that in patients between the ages of 15–49 with first-ever ischemic stroke, 13% had one or more SBIs. The study also found that 5% presented with leukoaraiosis and 3% presented with both. However, these numbers seem to increase with age. In the previously mentioned study, those between the ages of 15 and 24 did not present with SBIs or leukoaraiosis. The highest percentage of patients with SBIs or leukoaraiosis were those aged 45–49. Approximately 27% of patients had SBIs or leukoaraiosis, or both (17). Several studies also noticed an increase in the occurrence of SBIs and leukoaraiosis with age. A study conducted in Japan found that 57% of stroke patients with a mean age of 69, had SBIs (18). Similarly, our study found that patients with leukoaraiosis had a higher age, 55, compared to those without leukoaraiosis, 50. This is consistent was previously published studies which found that leukoaraiosis incidence increases with age (19, 20). Patients with SBIs also had a higher age when compared to those without.

Previous studies have shown various results regarding the incidence of SBIs in men compared to women. Generally, results of previous studies have shown that females were more likely to suffer from SBIs when compared to males (21). Two studies have found that females were 30%–40% more likely to suffer from SBIs than males (2, 11, 21).

Our study found that a higher percentage of males were more likely to have SBIs. A comparative study examined the relationship between sex differences in the risk profile and SBIs. It states that both brain infarction and SBIs were more common in males. However, after adjusting other cofounders, they found a difference in the occurrence of SBI occurrence males and females disappeared. Our study has also found no difference in the occupancy of leukoaraiosis between men and women. However, previous studies have shown that in stroke patients, women were more likely to have leukoaraiosis. In non-stoke patients, the difference was not established (22).

MetS refers to the combination of hypertension, diabetes, and obesity. Previous studies have shown that MetS is associated with both SBIs and leukoaraiosis (23–26). Our study has confirmed these results. 62% of the stroke patients included in the study suffered from MetS. Hypertension was found to be the most dominant component. The percentage was greater in patients with SBIs and leukoaraiosis. MetS was present in 87.5% of patients was SBIs and was also found in patients with leukoaraiosis at higher levels. Impaired fasting glucose and large waist circumference were also significantly associated with silent brain infarction. However, no association was found between large waist circumference and leukoaraiosis. This is consistent with other studies in the region, which showed that an association exists between MetS and leukoaraiosis. Elevated blood pressure and impaired fasting glucose were also independently associated. However, the large waste circumference was not (27). This study emphasizes the association between MetS and two specific conditions: leukoaraiosis and SBI. These conditions are likely caused by a common underlying vascular issue, namely atherosclerosis, which leads to small vessel disease (28). SBI was also found to be associated with a greater degree of leukoaraiosis.

SBIs and leukoaraiosis were also present in the general population but at fewer levels. A systematic review of published cohorts found that most studies have shown that SBIs occur between 10% and 20% in the general population (29). Similarly, it was found that the incidence of SBIs increases with age, with 35% of those over the age of 80 suffering from SBIs. The study also evaluated the effect of hypertension, dyslipidemia, and diabetes mellitus on the occurrence of SBIs. Hypertension was shown to impact the occurrence of SBIs and is considered one of the two most important risk factors. However, dyslipidemia and diabetes mellitus have shown various results. Leukoaraiosis incidence was also studied in the general population. It was found that 50.9% of healthy individuals between 44 and 48 had leukoaraiosis (30). The incidence also increases with age. Leukoaraiosis was found in 95% of people between the ages of 60–90. Although the pathogenesis of leukoaraiosis is unclear, it is known to be associated with dementia, stroke, abnormal gait, and disability (31). Leukoaraiosis is also used as an MRI marker for small vessel disease progression and is associated with worse stroke outcomes (32).

We recommend conducting longitudinal studies to explore the progression of SBI and leukoaraiosis over time in middle-aged stroke patients. Additionally, investigating the effectiveness of various treatment approaches, including lifestyle interventions and pharmacotherapy, in preventing or slowing down the development of these silent brain lesions would be valuable. Furthermore, assessing the association between silent brain lesions and long-term clinical outcomes, such as cognitive decline and recurrent stroke risk, is crucial for informing patient management strategies.

### Limitations

This study is subject to several limitations. Firstly, the sample size is relatively small, consisting of only 50 patients from a single center, which may limit the generalizability of the findings to larger populations. Another limitation is the potential oversight of clinically silent lacunes’ impact on cognitive performance, despite their significance in cerebral small vessel disease. Patients with a first-ever lacunar stroke often exhibit minor neuropsychological alterations related to these silent lacunar infarcts (33). Furthermore, this study employs a retrospective cohort design, which hinders the establishment of causal associations between variables. Future research should consider prospective study designs to better elucidate the relationships between various factors.

## Conclusion

SBI and leukoaraiosis observation have been made easier by developing newer imaging techniques, such as more advanced CT and MRI modalities. SBIs and leukoaraiosis were found to be associated with age and hypertension. They were also found in higher percentages in stroke patients compared to the general population. The relationship between SBIs, leukoaraiosis, and MetS is a complex relationship that requires further study in order to understand the underlying mechanism and provide better management, prevention, and treatment options. Through a better understanding of the underlying mechanisms of cerebrovascular disease, better clinical practices can be achieved which will provide better outcomes to all patients.

## Data Availability

The raw data supporting the conclusions of this article will be made available by the authors, without undue reservation.
